# *In vivo* efficacy of pitavastatin combined with itraconazole against *Aspergillus fumigatus* in silkworm models

**DOI:** 10.1128/spectrum.02666-23

**Published:** 2023-09-01

**Authors:** Hidetaka Majima, Teppei Arai, Katsuhiko Kamei, Akira Watanabe

**Affiliations:** 1 Division of Clinical Research, Medical Mycology Research Center, Chiba University, Chiba, Japan; 2 Department of Infectious Disease, Japanese Red Cross Ishinomaki Hospital, Ishinomaki, Japan; 3 Division of Infection Control and Prevention, Medical Mycology Research Center, Chiba University, Chiba, Japan; Universidade de Sao Paulo, Ribeirao Preto, Sao Paulo, Brazil

**Keywords:** statin, azoles, silkworm, *in vivo *efficacy, survival, histopathological analysis, qPCR, checkerboard, *Aspergillus fumigatus*

## Abstract

Azole resistance in *Aspergillus fumigatus* is a worldwide concern and new antifungal drugs are required to overcome this problem. Statin, a 3-hydroxy-3-methylglutaryl-coenzyme A (HMG-CoA) reductase inhibitor, has been reported to suppress the growth of *A. fumigatus*, but little is known about its *in vivo* antifungal effect against *A. fumigatus*. In this study, we evaluated the *in vivo* efficacy of pitavastatin (PIT) combined with itraconazole (ITC) against azole-susceptible and azole-resistant strains with silkworm models. Prolongation of survival was confirmed in the combination-therapy (PIT and ITC) group compared to the no-treatment group in both azole-susceptible and azole-resistant strain models. Furthermore, when the azole-susceptible strain was used, the combination-therapy resulted in a higher survival rate than with ITC alone. Histopathological analysis of the silkworms revealed a reduction of the hyphal amount in both azole-susceptible and azole-resistant strain models. Quantitative evaluation of fungal DNA by qPCR in azole-susceptible strain models clarified the reduction of fungal burden in the combination-therapy group compared with the no-treatment group and ITC-alone group. These results indicate that the efficacy of PIT was enhanced when combined with ITC *in vivo*. As opposed to most statins, PIT has little drug–drug interaction with azoles in humans and can be used safely with ITC. This combination therapy may be a promising option as an effective treatment in clinical settings in the future.

**IMPORTANCE:**

Azole resistance among *A. fumigatus* isolates has recently been increasingly recognized as a cause of treatment failure, and alternative antifungal therapies are required to overcome this problem. Our study shows the *in vivo* efficacy of PIT combined with ITC against *A. fumigatus* using silkworm models by several methods including evaluation of survival rates, histopathological analysis, and assessment of fungal burden. Contrary to most statins, PIT can be safely administered with azoles because of less drug–drug interactions, so this study should help us to verify how to make use of the drug in clinical settings in the future.

## INTRODUCTION

*Aspergillus fumigatus* is the predominant pathogen causing chronic pulmonary aspergillosis (CPA) and invasive pulmonary aspergillosis (IPA). A recent estimate suggests that more than 3 million subjects suffer from CPA worldwide, resulting in a mortality rate of at least 15%. As for IPA, from more than 200,000 cases, a greater than 50% mortality rate occurs each year (1, 2).

Only three classes of antifungal agents, azoles, echinocandins, and polyenes are available for the treatment of CPA and IPA. Azoles are their first-line antifungal drug therapy (3, 4). They can be administered both orally and intravenously and have fewer side effects compared to others (4). However, azole resistance among *A. fumigatus* isolates have recently been increasingly recognized as a cause of treatment failure (5, 6). It was reported that the prevalence of azole resistance ranges from 3.2 to 20% (5, 7). Azole resistance mechanisms are mostly correlated with mutations of cytochrome P450 sterol 14α-demethylase (Cyp51A), a target protein of azoles: the mutation of tandem repeats (TR) in promoter regions such as TR_34_/L98H and TR_46_/Y121F/T289A and point mutations in open reading frame regions at G54, G138, P216, M220, and G448 (8, 9). Mutations in 3-hydroxy-3-methylglutaryl-coenzyme A (HMG-CoA) reductase (*hmg1*) have recently been reported to be related to azole resistance (10 – 12). It is now widely known that some azole resistance can develop upon longer exposure to azoles at a sub-lethal concentration during the therapy of patients with aspergillosis (13, 14), although long-term oral antifungal therapy is recommended for pulmonary aspergillosis (2).

In order to overcome this dilemma, it is expected that safe, effective, and oral alternative antifungal drugs will be developed that are also effective for azole-resistant strains. However, the development of new drugs requires significant time and cost. Drug repositioning is a promising approach, and some studies have focused on the antifungal activity of non-antifungal drugs (15).

**Fig 1:**
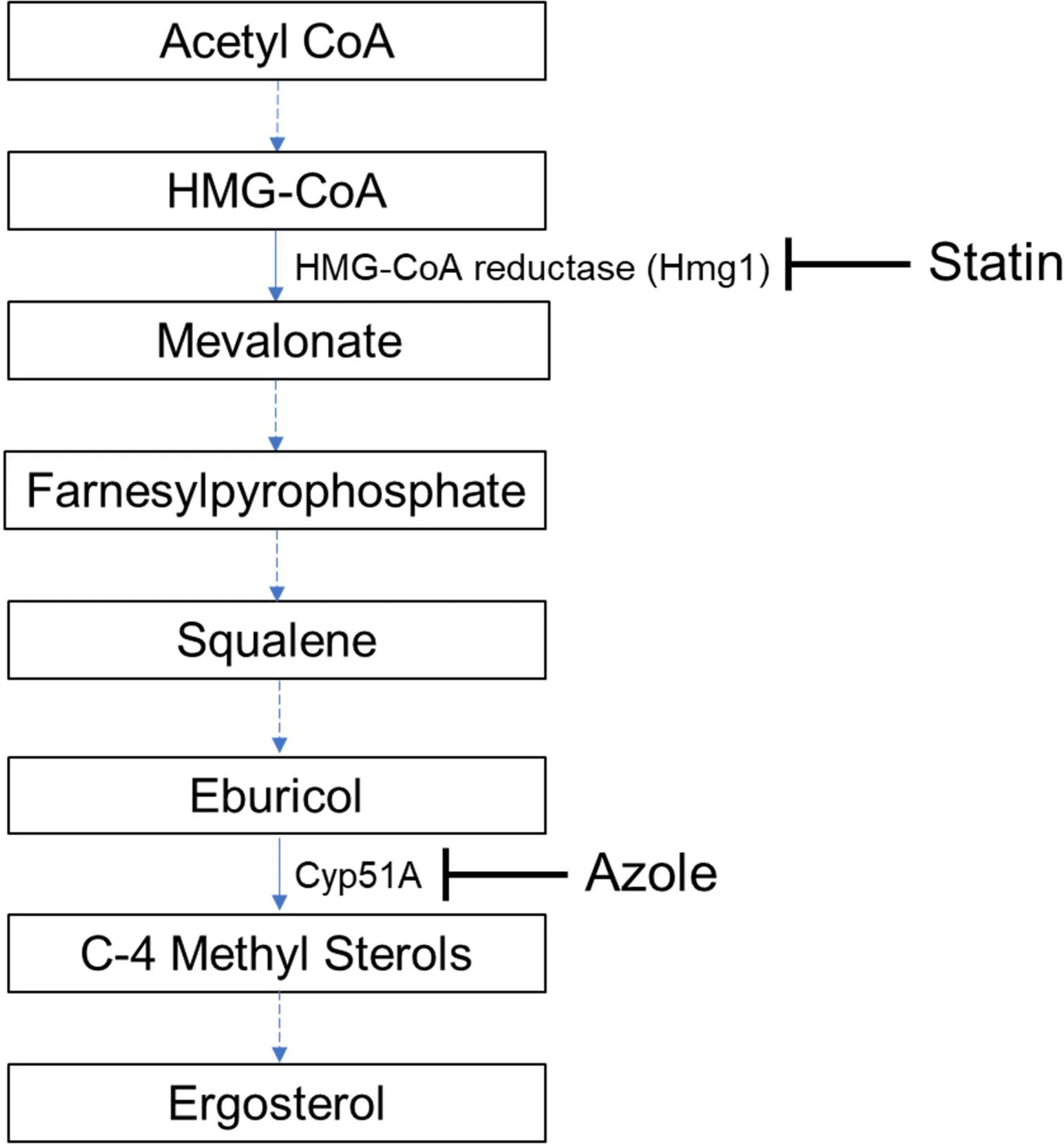
Ergosterol synthesis pathway in *Aspergillus fumigatus*. Azoles inhibit Cyp51 and statins inhibit Hmg1 on the ergosterol synthesis pathway.

Statins have also been explored for antifungal effect. They have been used for treating patients with dyslipidemia worldwide, since mevastatin was discovered as a metabolic product of *Penicillium citrinum* in 1976 (16). They show their cholesterol-lowering effect by inhibiting HMG-CoA reductase in humans, and they are thought to inhibit Hmg1 in fungi, which is also involved in the synthesis of ergosterol(Fig. 1) (17, 18). Lovastatin, simvastatin, and fluvastatin were reported to have an antifungal *in vitro* effect on *A. fumigatus* strains, including azole-susceptible strains (19, 20) and ITC-resistant strains (21).

Although their antifungal activity *in vitro* has been reported in many publications (17 – 22), it has been thought to be difficult to apply them *in vivo* because of their higher concentration than those available in human blood.

In addition, some reports confirmed the *in vitro* synergistic effect of the combination of statins and azoles: lovastatin and fluconazole against *Candida albicans* (23), and rosuvastatin, atorvastatin, or fluvastatin and azoles against *A. fumigatus* (22). However, another challenge for the *in vivo* applications is the drug–drug interaction. Azoles inhibit the cytochrome P450 enzyme (CYP3A4), which metabolizes most statins (24), making it more difficult to co-administer the statins and azoles in human bodies. These are some of the reasons why few studies have so far been reported concerning the *in vivo* antifungal activity of statin alone or statin and azoles.

Pitavastatin (PIT) and pravastatin are almost independent of CYP metabolism (25, 26), which makes it easier to combine them with azoles. As for its antifungal effect, Eldesouky et al. reported that PIT acts with fluconazole synergistically against some of *Candida* spp (27). On the other hand, little has been known about its antifungal activity against *A. fumigatus*, either *in vitro* or *in vivo*.

An *in vivo* study is especially an essential step for evaluating drug candidates or their derivatives, as we usually see the discrepancies between *in vitro* and *in vivo* activities. Recently, ethical issues have been raised regarding sacrificing mammals for the evaluation of the virulence of microbes or the efficacy of novel drugs, and especially those whose efficiency *in vivo* dose is not yet known (28). Silkworms, invertebrates, are experimental animals that can be used as alternative models for mammals. They have been reported as *in vivo* models for evaluating the pathogenecity of bacteria (29, 30) and fungi, including *C. albicans* (31), *Cryptococcus* spp. (32), *Trichosporon asahii* (33), and *A. fumigatus* (34). Moreover, pharmakokinetics and the toxity of many drugs are similar in silkworm models and mammal models (35 – 37). Nakamura also reported that silkworm models are useful for identifyng the candidates of effective antifungal agents against *A. fumigatus*, which were found to be effective in mouse models (38).

In this present study, we have evaluated the synergistic effect between PIT and azoles against azole-resistant and azole-susceptible *A. fumigatus* strains *in vitro*. Furthermore, we confirmed the *in vivo* antifungal effect of the combination therapy using silkworm models by several methods including evaluation of survival rates, histopathological analysis and assessment of fungal burden.

## RESULTS

### Evaluation of synergistic effect *in vitro* using checkerboard broth microdilution method

To determine the antifungal effect of PIT in combination with azoles, we examined azole-resistant isolates by checkerboard method. PIT alone showed an antifungal effect at 2–16 µg/mL (shown in Table 1). We also found a synergistic effect of PIT and ITC against 6 out of 10 azole-resistant strains. PIT showed a synergistic effect with ITC at a concentration of 1–4 µg/mL. We also confirmed a synergistic effect of PIT and voriconazole (VRC) against the isolates against which we found a synergistic effect of PIT and ITC (Table 2).

**TABLE 1:** Evaluation of synergistic effect *in vitro* between pitavastatin and itraconazole

Strain	Susceptibility to azoles	Genotype	MICs (µg/mL)				Interaction	
			PIT^*a*^ alone	ITC alone	PIT comb	ITC comb	FICI	Synergy
IFM60237	azole-resistant	*cyp51A* ^P216L^	4	>16	1	8	<0.75	NI
IFM63224	azole-resistant	*cyp51A* ^G448S^	4–8	16– > 16	0.5–1	2–4	<0.1875–0.3125	SYN
IFM63240	azole-resistant	*hmg1* ^S269F^	2–4	>16	0.5	1–2	<0.1875 - < 0.375	SYN
IFM63345	azole-resistant	*cyp51A* ^G54W^	8–16	>16	16	0	1	NI
IFM63432	azole-resistant	*cyp51A* ^TR46/Y121F/T289A^	4	2	2	0.25–0.5	0.625–0.75	NI
IFM63488	azole-resistant	*cyp51A* ^G448S^	8–16	4–8	0.25–4	1–2	0.313–0.563	NI
IFM63768	azole-resistant	*hmg1* ^S269Y^	8–16	16– > 16	1–2	1–2	<0.188–0.188	SYN
IFM64160	azole-resistant	*cyp51A* ^G448S^	4	8	1	1–2	0.188–0.5	SYN
IFM64304	azole-resistant	*cyp51A* ^M220V^	4	>16	0.5–1	2–4	0.375– < 0.375	SYN
IFM65548	azole-resistant	*cyp51A* ^M220I^	4	>16	1	2–4	<0.375– < 0.5	SYN
IFM62119	azole-susceptible	−	4–8	1	1–2	0.125–0.25	0.625–0.75	NI
IFM62153	azole-susceptible	−	4	1–2	1	0.25–0.5	0.5	SYN
IFM62234	azole-susceptible	−	4	1	2	0.0313–0.25	0.531–0.75	NI
IFM64301	azole-susceptible	−	4	1	0.5	0.5	0.625	NI
IFM64496	azole-susceptible	−	2	0.5–1	1	0.0313–0.25	0.581–0.75	NI
IFM64673	azole-susceptible	−	2–4	1	0.5–2	0.125–0.5	0.5–0.75	NI

^*a*^ PIT, pitavastatin; ITC, itraconazole; FICI, fractional inhibitory concentration index; NI, no interaction; SYN, synergism.

**TABLE 2:** Evaluation of synergistic effect *in vitro* between pitavastatin and voriconazole

Strain	Susceptibility to azoles	Genotype	MICs (µg/mL)				Interaction	
			PIT^*a*^ alone	VRC alone	PIT comb	VRC comb	FICI	Synergy
IFM60237	azole-resistant	*cyp51A* ^P216L^	4	1	1	0.25–0.5	0.5–0.75	NI
IFM63224	azole-resistant	*cyp51A* ^G448S^	8	16– > 16	2	1–2	0.313– < 0.375	SYN
IFM63240	azole-resistant	*hmg1* ^S269F^	2–4	4–8	0.5–1	0.5–1	0.313–0.5	SYN
IFM63345	azole-resistant	*cyp51A* ^G54W^	8	1	1–2	0.25–0.5	0.5–0.625	NI
IFM63432	azole-resistant	*cyp51A* ^TR46/Y121F/T289A^	4	>16	2	4	<0.75	NI
IFM63488	azole-resistant	*cyp51A* ^G448S^	16	16	4–8	2–4	<0.75	NI
IFM63768	azole-resistant	*hmg1* ^S269Y^	8–16	4	2–4	0.5	0.25–0.375	SYN
IFM64160	azole-resistant	*cyp51A* ^G448S^	4–8	16	1–2	0.5–2	0.281–0.375	SYN
IFM64304	azole-resistant	*cyp51A* ^M220V^	4	1	1	0.25	0.5	SYN
IFM65548	azole-resistant	*cyp51A* ^M220I^	4	1	1	0.25	0.5	SYN
IFM62119	azole-susceptible	−	8	1	1–2	0.125–0.25	0.375	SYN
IFM62153	azole-susceptible	−	4	0.5	0.5	0.125	0.375	SYN
IFM62234	azole-susceptible	−	4	1	1	0.25	0.5	SYN
IFM64301	azole-susceptible	−	4	0.5–1	1	0.125–0.25	0.5	SYN
IFM64496	azole-susceptible	−	2–4	0.5–1	0.5	0.125	0.375–0.5	SYN
IFM64673	azole-susceptible	−	4	0.5–1	1	0.125	0.375–0.5	SYN

^*a*^ PIT, pitavastatin; VRC, voriconazole; FICI, fractional inhibitory concentration index; NI, no interaction; SYN, synergism.

The checkerboard method was also performed for azole-susceptible strains. A synergistic effect was not shown in most azole-susceptible strains when PIT and ITC were combined (Table 1), but it was shown in all of the six azole-susceptible strains when PIT and VRC were combined (Table 2).

### Evaluation of survival rates

**Fig 2:**
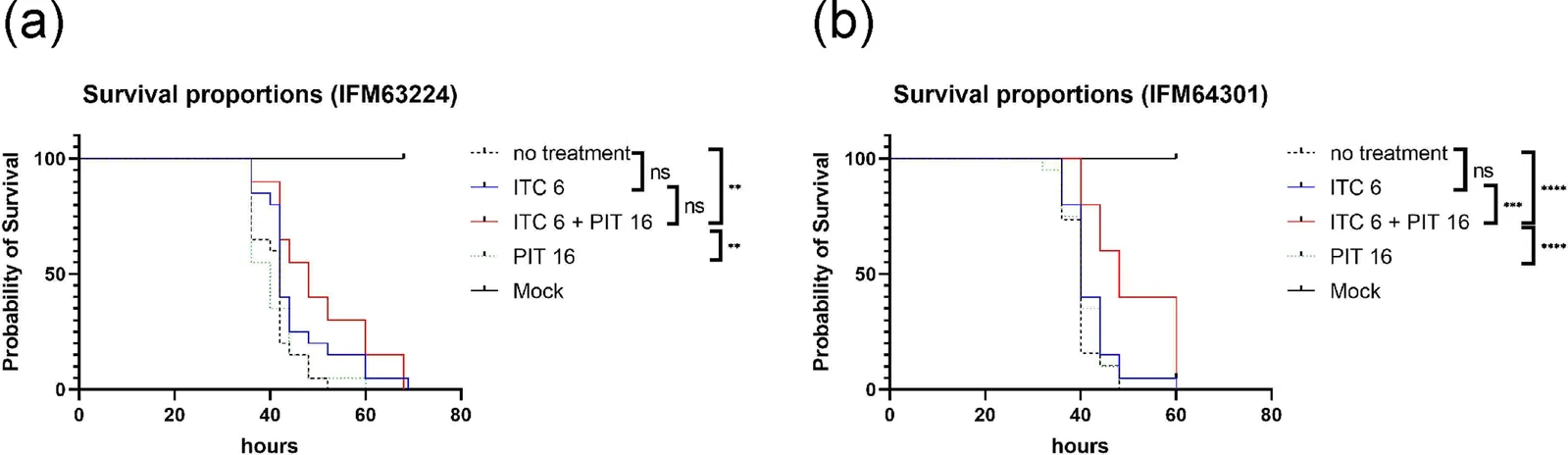
Evaluation of survival rates. The survival rates of silkworms were compared among no-treatment (*n* = 10), ITC-alone (*n* = 10), PIT-alone (*n* = 10), and a combination-therapy group (*n* = 10). We also made Mock (*n* = 5) as an uninfected group. The experiments were performed twice, and the results were shown as mean values. (**a**) Infection with azole-resistant strain (IFM63224). Combination therapy of PIT and ITC prolonged the survival compared to no treatment. (**b**) Infection with azole-susceptible strain (IFM64301). Combination therapy of PIT and ITC prolonged the survival compared to no treatment and ITC alone. Each symbol indicates the following: ns, *P* ≥ 0.05; *, *P* < 0.05; **, *P* < 0.01; ***, *P* < 0.001; ****, *P* < 0.0001.

In this experiment, antifungal agents were administered to the hemolymph of silkworms 24 h after infection with IFM63224, an azole-resistant strain harboring Cyp51A G448S substitution (inoculum of 5.0 × 10^6^ CFU/larva). We assessed the effect of PIT alone, ITC alone, and a combination of PIT and ITC on the survival of silkworms. As shown in Fig. 2, the survival rates in the combination-therapy (PIT and ITC) group was better than that in the no-treatment and PIT-alone groups, whereas no significant difference was detected between the combination-therapy and ITC-alone groups.

We also infected silkworms with IFM64301, an azole-sensitive strain with Cyp51A wild-type (inoculum of 2.5 × 10^5^ CFU/larva), followed by administration of antifungal agents to each group in the same way as above. Interestingly, the combination group showed prolonged survival compared to the no-treatment group, ITC-alone group, and PIT-alone group, although the combination therapy did not show any synergistic effect against this strain *in vitro* (Fig. 2).

### Histopathological analysis of silkworms

**Fig 3:**
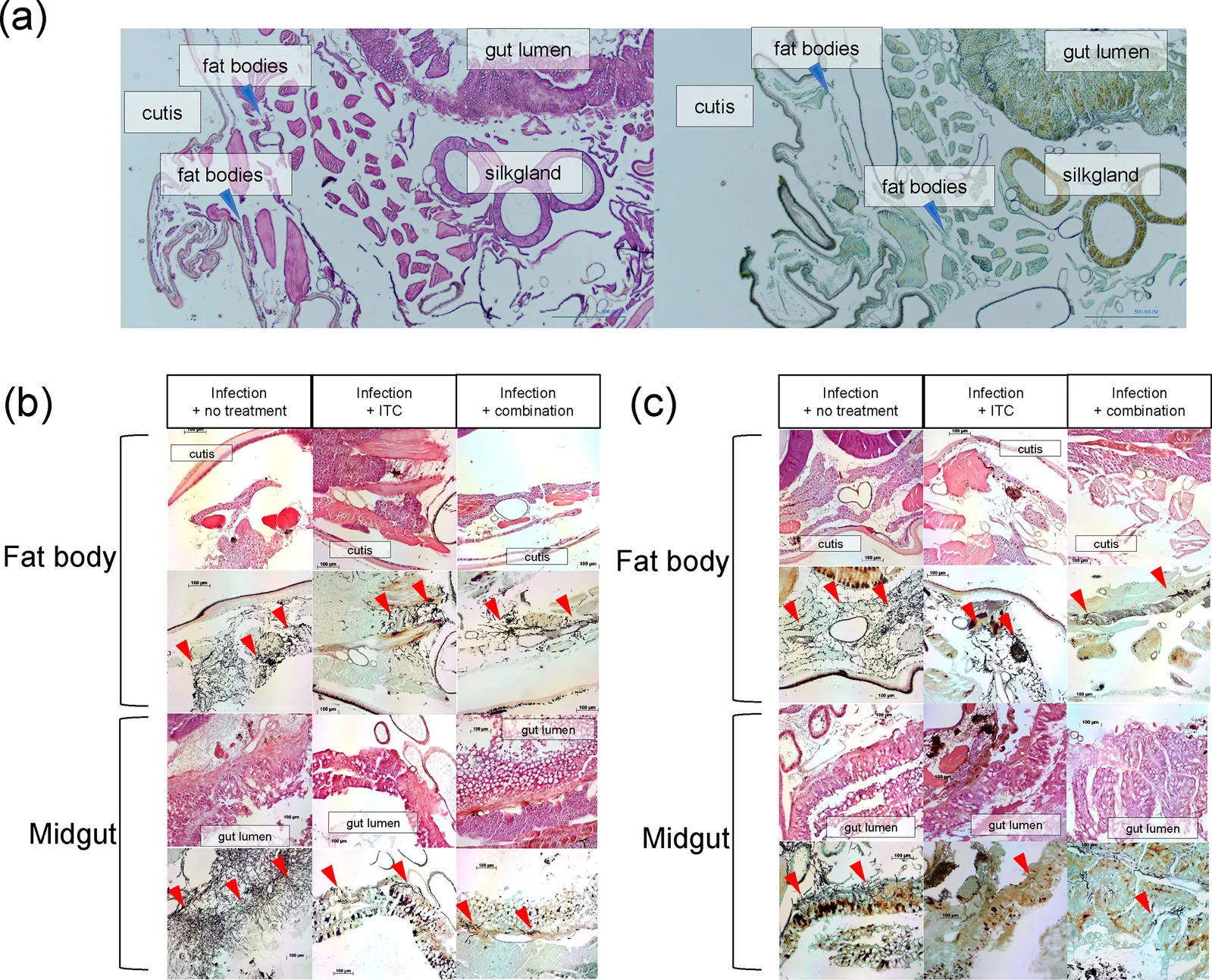
Histopathological analysis of silkworms infected with *A. fumigatus*. Thin sections were stained with hematoxylin-eosin staining and Grocott’s silver stain. (**a**) Normal histology in silkworm without infection. (**b**) Infection with azole-resistant strain (IFM63224). The amount of hyphae was reduced around the fat body and in the midgut of the combination-therapy group compared with the no-treatment group. (**c**) Infection with azole-susceptible strain (IFM64301). The amount of hyphae in the combination-therapy group was reduced around the fat body and in the midgut in comparison to the no-treatment group; it was also reduced around the fat body in comparison to the ITC-alone group.

We analyzed the histopathological findings due to treatments 32 h after infection in each strain by comparing them with those without infection as shown in Fig. 3a. After infection with the IFM63224 strain, which is azole-resistant, predominance of hyphae was determined around fat bodies in the no-treatment group, while it appeared that the amount of hyphae was reduced in the combination-therapy and ITC groups (Fig. 3b). The hyphal invasion to midgut was also suppressed in these two groups in comparison to the no-treatment group (Fig. 3b).

We also assessed the histopathology of silkworms infected with IFM64301, which is azole-susceptible. A reduction of the hyphal amount around fat bodies was observed in the combination-therapy group in comparison to not only the no-treatment group but also the ITC-alone group (Fig. 3c). The invasion of hyphae to the midgut was suppressed in the combination-therapy and ITC groups compared with the no-treatment group (Fig. 3c).

### Fungal burden assessment using qPCR

**Fig 4:**
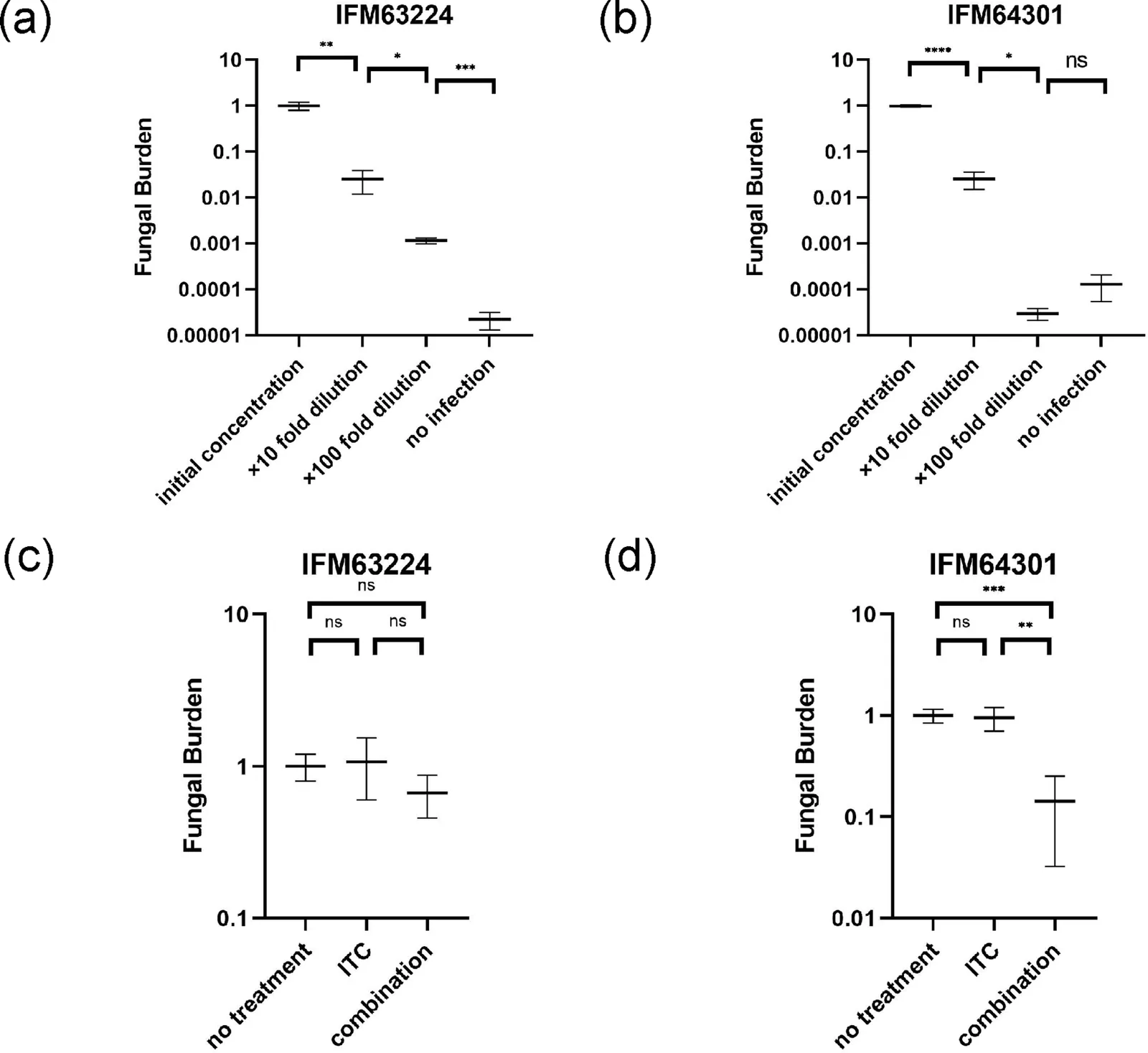
Evaluation of fungal burden using qPCR. (**a and b**) Fungal burden in whole bodies of silkworms was measured by qPCR, after infection with each diluted concentration of inoculum: initial concentration (*n* = 2), 10 × diluted concentration (*n* = 2), 100 × diluted concentration (*n* = 2). The initial concentration group (*n* = 2) was also assessed as controls. Fungal burden was confirmed quantitatively in an inoculum-dose-dependent manner by infection with IFM63224 (**a**) and IFM64301 (**b**). (**c and d**) Fungal burden was measured after infection and treatment: no-treatment (*n* = 2), ITC-alone (*n* = 2), combination-therapy (*n* = 2). (**c**) Infection with IFM63224. Combination therapy failed to reduce the fungal burden significantly compared with the no-treatment group. (**d**) Infection with IFM64301. Fungal burden in whole bodies was reduced significantly in the combination-therapy group compared with the no-treatment group and ITC-alone group. In the graphs (**a and b**) and (**c and d**), fungal burden of the initial concentration and no-treatment groups was taken as one, respectively. Mean values of two larvae in each group were shown. Bars indicate standard deviations. Each of the symbols indicate the following: ns, *P* ≥ 0.05; *, *P* < 0.05; **, *P* < 0.01; ***, *P* < 0.001; ****, *P* < 0.0001.

To further assess the efficacy of combination therapy *in vivo*, we measured and compared the fungal burden in each group. To establish the evaluation method of fungal burden, we needed to confirm whether the results of this method can be quantitively evaluated in an inoculum-dose-dependent manner. We measured the fungal burdens of silkworms inoculated with spore suspension at the same concentration as the survival test (initial concentration) as well as 10-fold and 100-fold diluted conidia suspensions, respectively. We could observe an increase of fungal burden according to the concentration of inoculum of each strain, IFM63224 and IFM64301, as shown in Fig. 4a and b, where the fungal burden of the group inoculated with an initial concentration was taken as one.

Second, we compared the fungal burden in each treatment group of silkworms infected with IFM63224. The mean value of fungal burden in the combination-therapy group was less than that in the no-treatment group, although the difference was not significant (Fig. 4c). In silkworm models infected with IFM64301, the fungal burden was reduced significantly in a combination-therapy group compared with the ITC-alone and no-treatment groups (Fig. 4d).

## DISCUSSION

In the present study, we confirmed that PIT combined with ITC is effective *in vivo* with silkworm models. To our knowledge, this is the first report to show the *in vivo* effect of statin combined with azoles against *A. fumigatus*. The results of our study suggest that this combination presents a potential new treatment strategy against *A. fumigatus*.

Contrary to most statins, PIT can be safely administered with azoles because of less drug–drug interactions. Statins have an antifungal effect *in vitro* (17 – 21), and the combinations of some statins and azoles have been reported to have synergistic effects *in vitro* (22, 23). These therapies have been expected as alternative antifungal drugs to overcome azole resistance in *A. fumigatus* isolates, which could lead to treatment failure of azoles. However, the combinations are difficult to be administered in clinical settings because of drug–drug interaction through CYP3A4 or CYP2A9, which leads to a higher risk of adverse events such as rhabdomyolysis (18, 39). PIT is mainly metabolized with glucuronidation and it has hardly any drug–drug interactions with azoles (25, 26). There has been a report that the human concentration of ITC is not influenced by PIT (40). The efficacy of PIT combined with ITC shown in the present study also suggests its potential as antifungal therapy.

In the present study, we confirmed its efficacy *in vivo* as well as *in vitro*. So far, some studies have reported the *in vivo* antifungal effect of statins. Tashiro et al. reported an *in vivo* antifungal effect of pravastatin against *C. albicans* in mouse models (41), and Eldesouky H.E. et al. confirmed the efficacy of PIT co-administered with fluconazole against *Candida* spp. in *Caenorhabditis elegans* models (27). In our study, we could find *in vivo* efficacy of the combination therapy against *A. fumigatus* including not only azole-resistant strain but also azole-susceptible strain, although the therapy was synergistic against only azole-resistant strain *in vitro*. We could not determine any apparent reason for this paradoxical finding. We often note a discrepancy between *in vitro* and *in vivo* activities of a targeted drug, as the *in vivo* therapeutic efficacy is affected by many factors such as the distribution and metabolism of the drug, as well as the *in vivo* virulence of pathogens. Further study is warranted, but we believe this study to be an important first step when we consider the application of statin or its analog to clinical drugs for humans.

Compared to the combination-therapy of PIT and ITC, PIT combined with VRC showed *in vitro* synergistic effect against more azole-susceptible strains. However, we could not find *in vivo* efficacy clearly when PIT was co-administered with VRC in silkworm models (data not shown). It seemed to be caused by the *in vivo* strong efficacy of VRC alone compared to ITC alone at a similar concentration. Therefore, we focused on the combination of PIT and ITC.

The silkworm, an invertebrate, has been expected as an *in vivo* model, and it has recently sometimes replaced mammal models in the field of infectious diseases, at least partly due to its lesser rearing cost and fewer ethical problems. This model enables us to evaluate the effects of antifungal drugs in a short period (28, 42). Furthermore, its metabolization of several drugs was shown to be similar to that of mammalian animals (37).

In our study, we could detect the efficacy of combination-therapy by other methods in addition to the evaluation of survival rates. First, histopathological analysis on silkworms infected with IFM64301 (azole-susceptible strain) in our study indicated the suppression of hyphal invasion to midgut in both the ITC-alone and the combination-therapy groups, and a reduction of hyphal amount around fat bodies only in the combination-therapy group. Although more investigation is needed, these results suggest that there is a drug distribution of antifungal agents in silkworm models, because the reduction of hyphal amount around fat bodies seemed to be caused by the combination-therapy, while the hyphal invasion to midgut seemed to be suppressed even by ITC alone. Detailed analysis of the pharmacokinetics in this model, including distribution, metabolism, and excretion, will give us a hint regarding the application of statin to mammal models.

Second, we also assessed the reduction of fungal burden using qPCR in a combination-therapy group. Since measurement using colony-forming units is not available for measuring the amount of filamentous fungi that grow in the form of multinucleated cells, the quantitative method using qPCR is a useful and objective way to measure the fungal burden in silkworm models. In our study, while the combination therapy reduced the fungal burden significantly compared to ITC-alone in silkworms infected with IFM64301, it failed to result in a significant difference compared to ITC-alone in those infected with IFM63224.

These results indicate that the combination therapy offered major efficacy against IFM64301, an azole-susceptible strain, rather than IFM63224, an azole-resistant strain, under the condition of our experiment. It is reported that azole-susceptible strains can acquire azole resistance caused during the lengthy administration of treating CPA (14). The antifungal effect on azole-susceptible strain should also be investigated because it may help as a precaution of azole-resistance acquisition. In addition, it seems that these methods will be efficient for evaluating other drugs in terms of several different perspectives.

The *in vitro* antifungal efficacy of PIT was exerted at relatively lower concentrations compared to other statins. Synthetic statins, including PIT, are usually considered to have more antifungal effect than fungal-derived statins (18). The effective concentrations of PIT combined with azoles were 0.5–8 µg/mL against several strains including azole-resistant ones *in vitro*. They were lower or similar to the synergistic concentrations of other synthetic statins, ATO (0.39 µg/mL) and ROS (12.5 µg/mL), combined with azoles against azole-susceptible strains (22, 41). However, the effective concentrations of PIT *in vitro* are still higher than those available as an antihyperlipidemic agent (Cmax: 0.1314 µg/mL in human body), which is mentioned in drug approvals and databases by the US Food and Drug Administration (https://www.accessdata.fda.gov/drugsatfda_docs/nda/2017/209875Orig1s000ClinPharmR.pdf) .

Furthermore, the effective dose (3 mg/kg larva body) in silkworm models is also higher compared to the dose of PIT for dyslipidemia in the human body (0.04 mg/kg body), although it is not easy to compare a one-time intra-hemolymph administration dose with a daily oral administration dose. For resolving this issue, a promising way would be to seek an optimization of the administration dose and timing, or to screen synthetic statins and their related analogs *in vitro* and *in vivo* using silkworm models, in which the effective doses of antimicrobial drugs were reported to be similar to those of mouse models and humans (35 – 37).

For the use of statins as antifungal drugs, it is also important to clarify the mechanism of the antifungal effect of PIT against *A. fumigatus*. Statins inhibit HMG-CoA reductase, which is a rate-limiting step in ergosterol synthesis. This is supported by the past report of the growth inhibition of *A. fumigatus* by simvastatin and atorvastatin being rescued when the media were supplemented with ergosterols (17). For *Saccharomyces cerevisiae*, it has been suggested that the reduction of ergosterol induced by statins increases the membrane penetration and helps another drug work inside the fungal cells when lovastatin is combined with azoles (43), and similar change of the membrane penetration may occur in *A. fumigatus*. Statins also have pleiotropic effects in fungal cells. They are thought to inhibit production of farnesyl pyrophosphate, which is an important intermediate metabolite in the synthesis of hemeA, ubiquinone, and prenylation of cellular proteins (Fig. 1) (18). Additionally, these mechanisms should also be assessed *in vivo* because it has been reported that the use of statin for lung transplant recipients is related to a lower risk of invasive aspergillosis in retrospective multivariable studies (44).

In conclusion, we elucidated the efficacy of PIT co-administered with azoles as an antifungal agent against *A. fumigatus in vitro* and *in vivo* for the first time. Determining the antifungal effect of PIT and its derivatives by *in vivo* silkworm models should help us to ascertain how to make use of the drug in clinical settings in the future.

## MATERIALS AND METHODS

### *A. fumigatus* strains

All clinical isolates were preserved at the Chiba University Medical Mycology Research Center (Chiba City, Japan), National Bio-Resource Project (NBRP) (http://www.nbrp.jp/), and were identified as *A. fumigatus* both by morphological characteristics and by sequencing the *β-tubulin* gene as previously described (45). For each experiment, strains were subcultured on Potato Dextrose Agar medium (Beckton Dickinson and Company, Sparks, MD, USA) and spores were harvested and adjusted to the concentration needed. All strains were investigated for *cyp51A* and *hmg1* mutations as previously described (12).

### Antifungal agents

ITC for checkerboard experiment and VRC for all experiments were purchased from Sigma (St Louis, MO, USA), and ITC for the other experiments and PIT were purchased from Fujifilm Wako Pure Chemical C (Osaka, Japan). All these drugs were purchased as standard powders and they were dissolved in dimethyl sulfoxide (DMSO).

### Antifungal susceptibility testing

We performed antifungal susceptibility tests using a broth microdilution method according to the Clinical and Laboratory Institute (CLSI) M38-Ed3 broth microdilution method (46), with partial modifications as described previously (47). Briefly, the inoculum was incubated with antifungal agents including ITC and VRC in RPMI 1640 medium (pH 7.0) at 35°C, using a dried plate for antifungal susceptibility testing (Eiken Chemicals, Tokyo, Japan). Strains with an elevated MIC of either ITC or VRC (≥4 µg/mL) were defined as azole-resistant and the others were considered azole-susceptible in this study. The experiment was performed in triplicate.

### Checkerboard broth microdilution method

The interactions between azoles and PIT were assessed by checkerboard method based on the CLSI M38-Ed3 protocol (46) with mild modifications, as previously described (48). Briefly, ITC or VRC (25 µL, 4-fold concentration) dilutions were prepared in the wells of 96-well round-bottom microtiter plates (Violamo, Osaka, Japan) with PIT (25 µL, 4-fold concentration) dilutions. The final concentration of agents against azole-resistant isolates ranged from 0.25 to 16 µg/mL for ITC or VRC, and from 0.25 to 16 µg/mL for PIT. On the other hand, the final concentrations against azole-susceptible isolates ranged from 0.0306 to 4 µg/mL for ITC or VRC and from 0.0613 to 4 µg/mL for PIT. Conidial suspension (50 µL) was added to each well to achieve a final inoculum size of 2.5 × 10^4^ CFU/mL. The plates were incubated at 35°C. Antifungal interaction was determined after 48 h by fraction inhibitory combination index (FICI) values expressed as follows:

FICI ≤ 0.5 indicates synergistic, 0.5 < FICI ≤ 4 indicates no interaction, and 4 < FICI indicates antagonistic. We conducted each experiment at least in triplicate.

### Silkworm infection experiments

Silkworm experiments were performed as described before, with slight modifications (31, 34, 38). Briefly, fifth-instar silkworms were purchased from Ehime Sansyu (Ehime, Japan) and were fed at 30°C for 2 days. Fifth instar day 3 larvae were infected with *A. fumigatus* strain using a 1-mL syringe equipped with a 29-gauge needle (Terumo Medical Corporation, Tokyo, Japan). We injected 50 µL of spore suspension adjusted to 5.0–10 × 10^6^ CFU/mL into their hemolymph, and this concentration of the spore suspension killed all the silkworms without any treatment within 48 h after infection. Then, 24 h after infection, we injected antifungal drugs into their hemolymphs. The stock solutions of PIT and ITC were diluted with DMSO, and were diluted with 0.6% NaCl to the designated concentrations in each experiment. We mixed fertilized PBS including red food coloring with spore suspension or antifungal agents to confirm the injections to their hemolymph.

### Evaluation of survival rates

We evaluated the effect of each antifungal agent up to 48 h after injection by comparing the survival rates of each group (no-treatment: *n* = 10; azole alone: *n* = 10; combination of PIT and azole: *n* = 10; PIT alone: *n* = 10; control without infection: *n* = 5).

### Histopathological analysis of silkworms

Larvae in each group (no-treatment: *n* = 2; ITC alone: *n* = 2; combination of PIT and azole: *n* = 2; control without infection: *n* = 2) were sacrificed after icing anesthesia at 32 h after infection and were cut sagittally at the middle of their bodies, the lower of which were conserved in formalin for 4 days. They were embedded in paraffin, and 3 µm thin sections were made and stained with hematoxylin-eosin and Grocott’s silver stain.

### Fungal burden assessment using qPCR

For estimation of fungal burden in infected larvae, we inoculated the larvae with the conidia suspension of each strain (50 µL) at the same concentration as in the survival experiment above, followed by administration of antifungal agents 24 h after infection.

Then, 32 h after infection, all of the larvae were sacrificed after anesthesia, cut into several pieces, and frozen at −80°C. The bodies of the larvae were homogenized with glass beads (5 mM and 0.5 mM) and DNA in the bodies were extracted by urea-phenol method (49). DNA pellets of each sample were diluted with 200 µL of distilled water. Then each DNA extraction was purified using the E.Z.N.A. fungal DNA kit (Omega Bio-Tek, Norcross, GA, USA) (50).

DNA quality and quantity were assessed with a NanoDrop 1,000 spectrophotometer (NanoDrop Technologies, Wilmington, DE, USA).

Fungal DNA was measured by real-time PCR on Applied Biosystems StepOnePlus (Thermo Fisher) by a modified method, with the TaqMan probe/primer set amplifying the 18S rRNA region (50) and IDT Prime Time Gene Expression (IDT Corporation, NJ, USA), according to the manufacturer’s instructions. Four-point standard curves were calculated with serial dilutions of each strain’s genomic DNA and the amount of fungal DNA was determined by calculation from the *C*_*T*_ value and the standard curve. To evaluate the correlation of inoculum-dose with fungal burden, we compared the fungal burdens among groups (initial concentration of inoculum: *n* = 2; 10 × diluted concentration: *n* = 2; 100 × diluted concentration: *n* = 2; no-infection: *n* = 2). For assessment of the combination therapy, we compared the fungal DNA of groups (no-treatment: *n* = 2; ITC-alone: *n* = 2; combination-therapy: *n* = 2; no-infection: *n* = 2).

For each test group, mean values of the fungal burdens of two larvae were evaluated.

### Statistical analysis

The survival rates of silkworms were assessed by Kaplan–Meier curves and the groups were each compared by log-rank test using GraphPad Prism 9 (GraphPad Software Inc. San Diego, CA, USA). The significance of differences in fungal burdens was compared by Welch’ s corrected *t*-test and a *P*-value < 0.05 was considered significant.
